# Renal dysfunction in fetal alcohol syndrome: a potential contributor on developmental disabilities of offspring

**DOI:** 10.12861/jrip.2014.24

**Published:** 2014-12-01

**Authors:** Farahnak Assadi

**Affiliations:** Section of Nephrology, Rush University Medical Center, Chicago, Illinois, UA

**Keywords:** Fetal alcohol syndrome, Developmental delay, Renal dysfunction

**Implication for health policy/practice/research/medical education:**

Alcohol consumption during pregnancy is teratogennic. The affects of prenatal alcohol exposure on offspring range from growth retardation, facial dysmorphism, and central nervous involvements. Impaired renal acidification has been documented in infants with fetal alcohol syndrome (FAS). The high urinary zinc excretion could deplete the zinc stores of the body leading to zinc deficiency. Zinc deficiency in pregnant mothers is also associated with fetal dysmorphogenesis. It is therefore possible that the increased zinc excretion in FAS is a spectrum of the impaired tubular dysfunction. The finding of low plasma zinc levels in infants with FAS suggest the possibility of preventing the alcohol-related birth defects by educating women of child bearing age to refrain drinking alcohol during pregnancy and by supplementing alcoholic pregnant women and their newborns with zinc. Further studies of zinc metabolism in alcoholic women and their neonates seem justified.

## Introduction

The term fetal alcohol syndrome (FAS) signifies the triad of intrauterine growth retardation, microcephaly and short palpebral fissures, due to prenatal exposure to alcohol (1-4). Those severely affected have unusual facial dysmorphism with microcephaly, narrow forehead, short palpebral fissures with hypoplastic upper lip (5-7). In addition, they have developmental delay, behavior dysfunction or deficit, intellectual impairment and/or mental retardation (8,9). The term fetal alcohol effects (FAE) describe less extreme changes associated with prenatal alcohol exposure. Criteria have not yet been established for the diagnosis of FAE, but there is consensus that the diagnosis requires deficiencies in two of the above categories and a history of maternal alcohol abuse (1-4).

Prenatal alcohol exposure has also been linked to cardiac anomalies, urinary and kidney anomalies, neural tube defects, cleft lip with or without cleft palate, gastrointestinal and genital abnormalities, dental anomalies, limb and joint abnormalities, scoliosis, ptosis, strabismus, epicanthal folds, microphthalmia, myopia, short up-turned nose, broad or low nasal bridge, low set ears and rotated ears (10-12).

Renal structural anomalies of FAS include cross-fused ectopia, renal hypoplasia and urethropelvic junction obstruction have been reported in children with FAS (10,12). More recent clinical and experimental studies have also shown that fetuses exposed to ethanol perinatally have a number of renal functional abnormalities, including impairment in renal acidification and potassium excretion as well as a defect in urinary concentrating ability, even in the absence of any structural abnormalities (13-15). The scanning electron microscopy examinations of the rat kidney following fetal exposure to alcohol, showed mitochondrial swelling and cytoplasmic vacuolation of the epithelial cells of the distal nephron, suggesting the possibility of alcohol induced renal tubular cell injury (16). Assadi and Ziai also reported that offspring of alcoholic mothers particularly those children with FAS have lower plasma zinc levels and that the urinary excretion of zinc is increased in these infants, suggesting a direct relationship between urinary losses and lower plasma zinc levels (17).

It is estimated that approximately 5% of all congenital birth defects are attributable to prenatal alcohol exposure across the globe (18-20). Estimates for the incidence of full-blown FAS, is 8 for every 1,000 live births, while that for FAE range from 10-30 per 1,000 live births (18-20). It is estimated that 110,000 newborns annually are affected by alcohol. Cost estimates for FAS/FAE are exorbitant. It is also estimated that 12% of the annual cost for all institutional care for the mentally retarded patients, in the Western communities, are for individuals with FAS/FAE (21).

Despite the wide spread incidence, FAS remains to be difficult to diagnose clinically, and difficult to research. The clinical features may not be readily obvious and individually are not specific to only FAS (6,9,22). The neonatal period is the most difficult to diagnose FAS. In the neonate the facial dysmorphic feature is subtle, often obscured by periorbital or general facial edema. Mental retardation and developmental impairment may not be evident for several years. Throughout gross specific clinical features can vary. No single feature is found in every case. Also, there exists some overlap between FAS and other syndromes. A history of alcohol abuse in pregnancy is often under reported or denied. Physicians tend to under diagnose FAS either due to lack of familiarity with the syndrome or a reluctance to place a label with negative social connotations.

The most distressing consequence of prenatal alcohol exposure is the central nervous system damage (7,8). The affected newborn may exhibit poor sucking, tremulousness, unusual body posturing, abnormal reflexes, and disturbed sleep patterns. The older infant demonstrates measurable decrements in mental and motor development, poor visual recognition, memory and decreased expressive and receptive language. Affected preschoolers demonstrate decreased IQ scores, attention deficits, delayed reaction time and decrements in fine and gross motor performance. The school aged child has decreased achievement scores, is easily distracted, learning disabled, shows poor organization and cooperation and has a rigid approach to problem-solving (7,8). Adolescents and adults with FAS have an average IQ of 65 with a range from 20 to 90. Those with FAE have an average IQ of 80, ranging from 40 to 100 (1-6). Despite higher IQ scores, individuals with FAE generally have achievement test scores similar to those with full FAS. In general, more physical manifestations are associated with poorer intelligence. However, there is a wide overlap in the ranges and the degree of brain damage is not always directly proportional to the severity of physical malformations, making it difficult to predict intelligence on an individual basis. Adolescents and adults exhibit a wide variety of behavioral problems, including impulsivity, social withdrawal and anxiety. Most have problems in adaptive living, lacking judgment and problem-solving skills. Few adults with FAS or FAE are self-sufficient.

The identification of renal dysfunction and zinc deficiency in FAS is important from a prognostic point of view and to permit adequate and comprehensive management of patients with this syndrome.

Previous studies have shown that birth defects that result from prenatal zinc deficiency are likely caused by a defect in DNA and protein synthesis (23-25). It is also known that zinc deficiency can lower the activity of alcohol dehydrogenase in multiple organs of alcoholic patients (26).

Because zinc plays an important role in DNA synthesis and current data are inconclusive, it is possible that alcohol-induced zinc deficiency may contribute to alcohol related birth defects observed in FAS (17). This possibility remains a reasonable hypothesis and requires further study.

Alcohol induced organ damage arises not only from the direct effects of alcohol on tissues but also from the metabolism and the metabolites of alcohol. Recent investigations have focused on assessing the direct effect of alcohol versus acetaldehyde toxicity, the role of nutrition with impaired placental function and synergic effects of nicotine and caffeine (26). More specifically, mechanisms for FAS involving altered protein synthesis and hypoxemia have been considered (26). Class I and Class II alcohol dehydrogenases are the enzymes that are primarily responsible for initial degradation of alcohol, acetaldehyde, in humans. It has been found that acetaldehyde can cross the human placenta from the maternal to the fetal compartments. Toxic aldehydes generated in this initial degradation step are further oxidized by aldehyde dehydrogenases. It seems likely that genetic variation in alcohol metabolism may lead to differences in the propensity of individuals to develop alcohol induced organ damage. With DNA probes, it will now be possible to assess the role of alcohol metabolizing enzymes in the development of organ damage.

As to a specific mechanism by which alcohol may impair fetal growth and development, impaired protein synthesis, abnormal delivery or regulation of glucose in the fetus, altered zinc metabolism, and hypoxia are key considerations under investigation. Better understanding of the mechanism involved may permit more meaningful therapeutic intervention.

The timing of alcoholic exposure is also crucial (22). The most crucial period for anatomic abnormalities is early in the first trimester, which includes the time segment preceding pregnancy recognition. The third trimester appears to be the most vulnerable period for overall gross and central nervous system development. Intrauterine growth retardation cannot be detected by ultrasonography until approximately 27 weeks gestation.

FAS/FAE is particularly tragic because it is totally preventable, simply through abstinence from alcohol during pregnancy. It is the responsibility of health care professionals, social and government agencies to protect the unborn child from the devastating effects of alcohol. The most effective weapon for prevention of FAS is education which targets every non-pregnant of childbearing age (27,28). Pregnant women who are identified to use alcohol should be intensively counseled to discontinue further alcohol consumption. Although this will not reverse any damage already produced, at least any further damage is prevented.

Identification of women at risk is often difficult. Simple questioning can be unreliable because of poor recall or denial. Since direct questioning regarding alcohol can be intimidating, questionnaires may be developed for assessment of risk. Hopefully through continued educational efforts, we will successfully imprint the awareness that alcohol consumption during pregnancy is unacceptable.

## Conclusion

Universal health care prevention is urgently needed to educate women of reproductive age about the increased risk of alcohol-related birth defects. Because FAS/FAE is entirely preventable, avoiding alcohol consumption during pregnancy should be the first attempt to reduce the increased risk of birth defects. Renal evaluations of neonates born to alcoholic mothers are indicated for the early diagnosis. Management of correctable abnormalities that might contribute to child growth and developmental delay, including zinc supplement, should also be considered.

## Author’s contribution

FA is the single author of the manuscript.

## Ethical considerations

Ethical issues (including plagiarism, informed consent, misconduct, double publication and redundancy) have been completely observed by the author.

## Conflict of interests

The author declared no competing interests.

## Funding/Support

None.
